# Comparing the effects of verbenone, methyl salicylate, and permethrin on attacks of ambrosia beetles (Coleoptera: Curculionidae Scolytinae) in ornamental nurseries

**DOI:** 10.1093/jisesa/ieaf008

**Published:** 2025-01-27

**Authors:** Ramkumar Govindaraju, Shimat V Joseph

**Affiliations:** Department of Entomology, University of Georgia, Griffin, GA USA; Department of Entomology, University of Georgia, Griffin, GA USA

**Keywords:** phytochemical, specialty crop, semiochemical, tree protection, pyrethroid

## Abstract

The granulate ambrosia beetle, *Xylosandrus crassiusculus* (Motschulsky), and the black stem borer, *Xylosandrus germanus* (Blandford) are important pests in ornamental nurseries in the eastern USA. These beetles are managed mainly using preventative trunk applications of pyrethroids, such as permethrin or bifenthrin when females typically fly out of woodlots and attack young trees in the spring. Verbenone and methyl salicylate are potential phytochemicals reported as repellants but not completely validated in ornamental nurseries for ambrosia beetle management as an alternative option. Thus, this study aimed to compare the efficacy of verbenone alone or with methyl salicylate and permethrin on ambrosia beetle attacks. In 2023 and 2024, a study was conducted where verbenone (with methyl salicylate) and permethrin were combined with 10% and 50% ethanol-infused maple bolts. Verbenone alone or combined with methyl salicylate did not reduce ambrosia beetle attacks on bolts compared to permethrin. Permethrin was effective in reducing ambrosia beetle attacks on bolts. This suggests that permethrin is still the best option to prevent ambrosia beetle attacks on young trees in ornamental nurseries.

## Introduction

The granulate ambrosia beetle, *Xylosandrus crassiusculus* (Motschulsky), and the black stem borer, *Xylosandrus germanus* (Blandford) (Coleoptera: Curculionidae: Scolytinae) are serious ambrosia beetle pests of ornamental nurseries (Ranger et al. 2016, Gugliuzzo et al. 2021, Monterrosa et al. 2022). In the spring, mated females of *Xylosandrus* spp. typically fly out of woodlots in large numbers and attack young, stressed trees in ornamental nurseries. The stressed trees release ethanol, and females use it as a cue to locate suitable hosts to colonize (Ranger et al. 2010, 2015). Then, females bore through the bark and make galleries inside the xylem in the center of the tree (Ranger et al. 2016), where they farm the symbiotic fungi they store in a specific organ called a mycangium (Weber and McPherson 1983). While digging galleries, females produce “noodle”-shaped compressed sawdust that protrudes from entry holes (Ranger et al. 2016). Affected trees develop branch dieback, and some die (Ranger et al. 2016). These nonsalable, damaged trees, and the cost of management accumulate several million dollars in losses to the nursery industry, highlighting the serious financial impact of ambrosia beetle infestations and the need to develop effective management strategies (LeBude et al. 2012).

In ornamental nurseries, ambrosia beetles are managed using pyrethroids, especially permethrin and bifenthrin (Ranger et al. 2016). Growers spray pyrethroids to prevent invading ambrosia beetles from attacking trees, especially early in the spring, before and during bud break (Frank and Sadof 2011, Reding et al. 2013, Ranger et al. 2016). However, pyrethroid application does not guarantee a consistent reduction in ambrosia beetle attacks (Hudson and Mizell 1999, Frank and Sadof 2011, Ranger et al. 2016, Monterrosa et al. 2024). In the spring, trees, such as maple (*Acer* spp.), redbud (*Cercis* spp.), and so on, produce flowers that attract pollinators, such as bees, moths, flies, and so on. Thus, the timing of pyrethroid applications intended to protect trees from ambrosia beetle attacks coincides with the flowering of trees in ornamental nurseries, posing the potential risk of pollinator exposure to these chemicals (Frank and Sadof 2011). This ethical consideration further underscores the need to determine alternative approaches to mitigate the risk, such as reduced-risk insecticides or other sustainable approaches.

Verbenone is widely reported as a repellent for scolytine beetles, including *Xylosandrus* spp. (Ranger et al. 2013, Rivera et al. 2020, Agnello et al. 2021, Lindgren and Miller 2022, Frühbrodt et al. 2024). Verbenone, a monoterpene ketone with 2 enantiomers, is naturally biosynthesized through the oxidation of verbenol in conifers and angiosperms (Frühbrodt et al. 2024). It was first recorded in the mountain pine beetle, *Dendroctonus ponderosae* Hopkins, in western North America (Pitman et al. 1969) where females use verbenone after oviposition to signal conspecific females discouraging from oviposition (Pitman et al. 1969). In addition, scolytine beetles metabolize α-pinene into cis- and trans-verbenonol upon ingestion, and the yeast in the gut converted it to verbenone (Leufvén et al. 1984, Borden et al. 2003). Verbenone reduces *D. ponderosae* attacks in lodgepole pine *Pinus contorta* Dougl. ex Loud. stands (Fettig and Munson 2020). Similarly, the effects of verbenone on the southern pine beetle, *Dendroctonus frontalis* Zimmermann were evaluated and have shown promise (Sullivan and Clarke 2021). For the western pine beetle, *Dendroctonus brevicomis* LeConte management, verbenone alone elicited a limited reduction in attacks on ponderosa pine, *Pinus ponderosa* Douglas ex Lawson (Fettig et al. 2009). Interestingly, verbenone plus, a combination of acetophenone, (*E*)-2-hexen-1-ol + (*Z*)-2-hexen-1-ol, and (–)-verbenone reduced *D. brevicomis* attacks on *P. ponderosa* (Fettig et al. 2012). Verbenone was tested as a pest management tactic also in tree fruit orchards (Rivera et al. 2020, Agnello et al. 2021) and in ornamental nurseries (Ranger et al. 2013, Williamson et al. 2023). When trees were closer to the verbenone source, ambrosia beetle attacks were reduced (Ranger et al. 2013). Similarly, trap captures of the redbay ambrosia beetle, *Xyleborus glabratus* Eichoff, were reduced when verbenone was used on sticky traps (Hughes et al. 2017, Martini et al. 2020). Methyl salicylate is another volatile compound produced through the salicylic acid pathway in plants (Gondor et al. 2022). Methyl salicylate enhances systemic acquired resistance (SAR) and could increase insect resistance and repellency (Gao et al. 2015). When combined with verbenone, it was shown to reduce attacks of *X. glabratus* on redbay (*Persea borbonia* [L.]; Lauraceae) (Hughes et al. 2017) and *X. germanus* attacks on apple (*Malus domestica* Borkh; Rosaceae) (Agnello et al. 2021).

Although verbenone was directly compared with permethrin against ambrosia beetles (Williamson et al. 2023), no study has yet directly compared the efficacy of verbenone and methyl salicylate vs. permethrin. Thus, this study aimed to determine the efficacy of verbenone, methyl salicylate, and permethrin on bolts in ornamental nurseries.

## Materials and Methods

### Study Site

In the spring of 2023 and 2024, this study was conducted in a 101.2 ha ornamental nursery in Pike County (33.04071061170671, -84.3354113471871), Georgia, USA. The ornamental nursery was surrounded by a mixed hardwood pine woodlot with a variety of trees and shrubs, such as sweetgum (*Liquidambar styraciflua* L.), maple, oak (*Quercus* spp.), pine (*Pinus* spp.), and privet (*Ligustrum* spp.). In the nursery, a variety of woody ornamental trees, such as magnolia (*Magnolia virginiana)*, oak, maple, and so on, were grown, and they were 1 to 3 yr old. These young trees were under drip irrigation and received routine pruning and fertilization throughout the growing season. Insecticides were not used in the nursery block where the study was conducted for the duration of the experiment in both years.

### Chemicals

Two repellents were used in the study. They were verbenone (Verbenone, Synergy Shield Verbenone pouches 97.0%; Cat#3562; Synergy Semiochemical Corporation, Delta, British Columbia, Canada) and methyl salicylate pouch (methyl salicylate 100%; Cat#3512; 10 mil pouch; Synergy Semiochemicals Corp, Delta, BC, Canada). A pouch with (-) verbenone (4, 6, 6-trimethyl-bicyclo (3.1.1) hept-3-en-2-one) had 6 ml load with a release rate of 40 mg per day at 25 °C, whereas a methyl salicylate pouch had 4 ml load with a release rate of 100 to 125 mg per day at 25 °C. The manufacturer recommends field deployment of approximately 90 d for the verbenone pouch. The insecticide used in the study was permethrin (Perm-UP 3.2EC; FMC Philadelphia, PA, USA). The rate of Perm-UP 3.2EC is 584 ml per ha. The water volume used in both years was 935.4 L per ha. The insecticide solution was sprayed on the bolts using the CO_2_-powered sprayer at 206.843 kpa before deployment.

### Experimental Design

The experiment was conducted using red maple (*Acer rubrum* L.) bolts. Red maple branches were obtained from the residential yards in Pike County, Georgia, USA. From red maple branches, 30 cm (long) × 6 cm (diam.) bolts were prepared. A 10 cm (deep) × 1.2 cm (diam.) vertical core was drilled on each bolt at the center. The hole was then sealed using a cork. The bolt was suspended approximately 0.61 m above ground using a metal stake. In 2023, 10 ml of 10% and 50% ethanol was filled in the cores, whereas in 2024, only 10% ethanol was used to fill the core. The experiment was conducted twice and was initiated on 30 March 2023 and 27 February 2024. Every week, 10 ml ethanol was added to refill the core of each bolt.

Ethanol concentrations ≥ 50% were used in previous studies (Frank and Sadof 2011, Agnello et al. 2021, Williamson et al. 2023). The effects of verbenone and permethrin were tested under 2 concentrations of ethanol (10% and 50%) in the first year; then, the 50% ethanol treatments were excluded from the experiment as a recent study showed that 10% ethanol release from bolts was comparable to concentrations of ethanol naturally released from stressed trees (Ranger, unpublished data). In the second year, methyl salicylate pouches were added with verbenone pouches to enhance repellency effects to reduce ambrosia beetle attacks, as reported previously by Hughes et al. (2017), Rivera et al. (2020) and Agnello et al. (2021).

In 2023, treatments were (i) no ethanol [0%EtOH]; (ii) 10% ethanol (10%EtOH); (iii) 10%EtOH + Verbenone [V]; (iv) 10%EtOH + Permethrin [P]; (v) 50% ethanol (50%EtOH); (vi) 50%EtOH + V; and (vii) 50%EtOH + P. In 2024, treatments with 50% ethanol were excluded, and methyl salicylate (M) was added to treatments that had verbenone. Thus, treatments were (i) 0%EtOH; (ii) 10%EtOH; (iii) 10%EtOH + VM; (iv) 10%EtOH + P; and (v) 10%EtOH + VM + P. In both years, treatments were arranged in a randomized complete block design with 6 replications. Verbenone and methyl salicylate pouches were deployed only once at the beginning of the experiment and maintained for the duration of the study (not replaced). Verbenone and methyl salicylate pouches were secured on the bolts using a screw. For those treatments where permethrin was sprayed, verbenone and methyl salicylate pouches were immediately attached after permethrin was sprayed on bolts. Similarly, permethrin was sprayed only once on the bolt at the beginning of the experiment (0 d). Bolts were deployed 10 m apart along the wood line (Williamson et al. 2023 and, Govindaraju et al. 2025) about 0.5 m inside the nursery from the tree line. Bolts were exposed to ambrosia beetle attacks for 3 wk.

### Evaluation

The number of entry holes on bolts was marked (circled) with colored markers every week and counted. The bolts were evaluated at 7, 14, and 21 d post-deployment on 6, 13, and 20 April 2023 and 5, 12, and 19 March 2024, respectively. At the end of the trial, all bolts were collected, placed in plastic bags, and transported to the laboratory. It is possible that some ambrosia beetles left the bolts immediately after the attacks, a phenomenon commonly occurring when the substrate is not suitable (Biedermann et al. 2009, Cruz et al. 2018), and that we might have underestimated the abundance of certain species inside the wood. To determine the ambrosia beetle species in the bolts, bolts were split 8 ways using a hammer, and the fallen ambrosia beetles were quantified. Because the study aimed to determine the attack patterns rather than colonization success, bolts were slit open to recover females within 2 wk after bolts were brought to the laboratory. Thus, the females collected from bolts were mostly foundresses. They were stored in 70% ethanol and later identified to genus and species using a lucid key designed for species of Southeast Asian Xyleborini (Bateman and Hulcr 2017, Smith et al. 2019).

### Statistical Analyses

The total entry hole data across 6 replicates were pooled by treatment. Adult ambrosia beetles recovered from bolts were also pooled across 6 replicates by treatment and analyzed as total ambrosia beetles, *X. crassiusculus* and *X. germanus*. The entry hole and ambrosia beetle data were subjected to a 1-way analysis of variance using a generalized linear model (PROC GLIMMIX) in SAS (SAS Institute 2024). The model was set with a log-link function and using a Poisson distribution. The treatment and replication (block) were fixed and random effects, respectively, in the model. For entry hole data, a value of one was added as data were zero-inflated in 2024. The means were separated by the Tukey–Kramer test (α < 0.05). Means and standard errors were calculated from non-transformed data using the PROC MEAN procedure in SAS.

## Results

In 2023, of 199 beetles recovered from the bolts, 146, 32, 4, and 17 were adult *X. crassiusculus*, *X. germanus*, *Xylosandrus compactus* (Eichhoff), and other scolytine beetles, respectively. There were 287 entry holes on all bolts combined. In 2024, of 29 beetles recovered, all of them were adult *X. crassiusculus,* and no adult *X. germanus* and *X. compactus* were collected. There were 106 entry holes on all bolts combined.

**Fig. 1.**
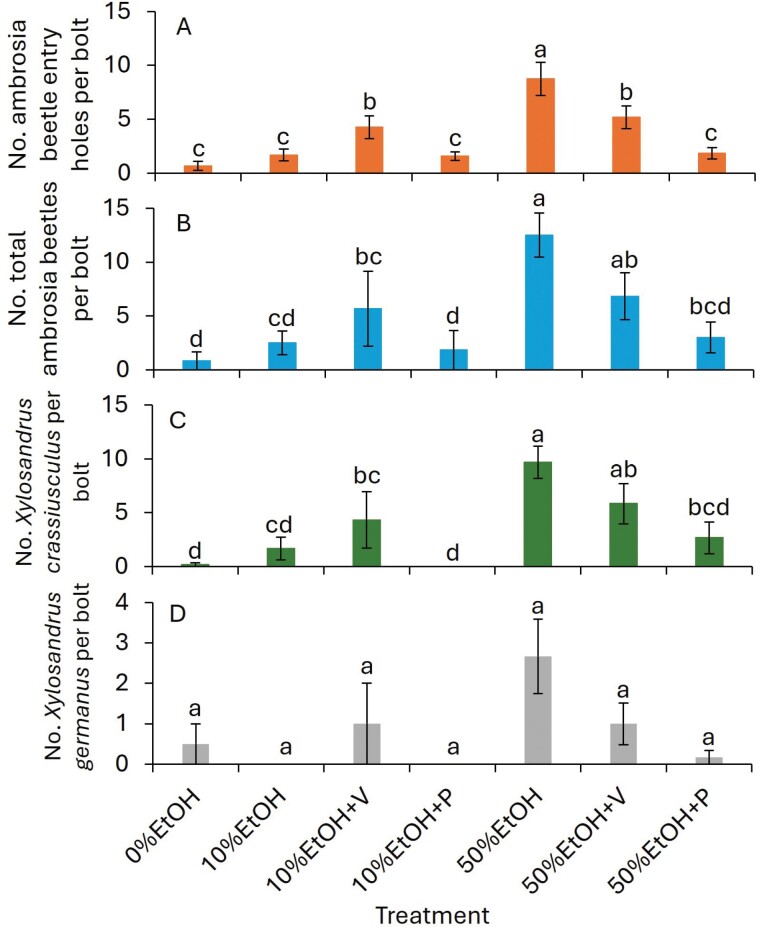
Mean ± (SE) number of (A) entry holes, (B) total adult ambrosia beetles, (C) adult *Xylosandrus crassiusculus*, and (D) adult *X. germanus* collected in 3 wk after exposing 10% and 50% ethanol-infused bolts in the ornamental nursery in 2023. The abbreviations were EtOH, ethanol; V, verbenone; M, methyl salicylate; and P, permethrin. The same letters above bars indicate no significant difference using the Tukey-Kramer test (α < 0.05).

In 2023, the number of entry holes was significantly greater for the 50% ethanol-only treatment than for the 10%EtOH + V and 50%EtOH + V treatments followed by the remaining treatments (*F* = 22.5; df = 6, 30; *P* < 0.001; Fig. 1A). There were no significant differences between 0%EtOH, 10%EtOH, 10%EtOH + P and 50%EtOH + P treatments. The numbers of total adult ambrosia beetles and adult *X. crassiusculus* were significantly more for the 50%EtOH treatment than for the 10%EtOH + V treatment followed by the 0%EtOH and 10%EtOH + P treatments (Total ambrosia beetles: *F* = 15.9; df = 6, 30; *P* < 0.001; Fig. 1B; *X. crassiusculus*: *F* = 12.7; df = 6, 30; *P* < 0.001; Fig. 1C). The number of adult *X. germanus* was significantly different (*F* = 2.8; df = 6, 30; *P* = 0.029; Fig. 1D) but Tukey–Kramer test did not separate treatments at α < 0.05.

**Fig. 2.**
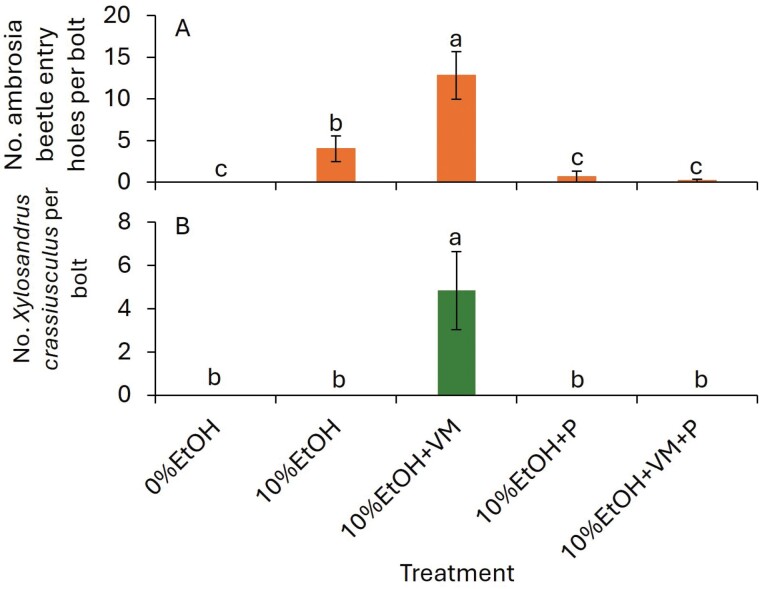
Mean ± (SE) number of (A) entry holes and (B) adult *Xylosandrus crassiusculus* collected in 3 wk after exposing 10% ethanol-infused bolts in the ornamental nursery in 2024. The abbreviations were EtOH, ethanol; V, verbenone; M, methyl salicylate; and P, permethrin. The same letters above bars indicate no significant difference using the Tukey-Kramer test (α < 0.05).

In 2024, the number of entry holes was significantly greater for the 10%EtOH + VM treatment than for the 10%EtOH treatment followed by the remaining treatments (*F* = 26.8; df = 4, 20; *P* < 0.001; Fig. 2A). The number of adult *X. crassiusculus* was significantly greater for the 10%EtOH + VM treatment than for the remaining treatments (*F* = 11.1; df = 4, 20; *P* < 0.001; Fig. 1B). Adult *X. germanus* and other adult ambrosia beetle were not captured in the 2024 experiment.

## Discussion

In this study, we sought to validate the effect of verbenone further and determine how it compares with permethrin trunk application so that a pollinator-friendly management approach can be developed as part of an integrated pest management program in ornamental nurseries. Results showed that verbenone did not reduce ambrosia beetle attacks on bolts compared to permethrin. This result is consistent with Williamson et al. (2023), where data showed that verbenone reduced the number of approaching ambrosia beetles but did not reduce the attacks. In Williamson et al. (2023), a 90% ethanol concentration was used, which is however not comparable to the real ethanol content in stressed trees. In stressed ornamental trees, approximately 10% ethanol is released (Ranger unpublished data). Thus, in the current study, 10% and 50% ethanol concentrations were initially used, and then 50% ethanol concentration was removed, considering the recent unpublished study. Using 10% ethanol concentration, ambrosia beetle attacks were low, and attacks were greater on bolts with verbenone than without. Moreover, methyl salicylate was combined with verbenone in the 2024 experiment, but the combination of the 2 lures did not reduce ambrosia beetle attacks either. It is unclear why verbenone and methyl salicylate attracted more ambrosia beetle adults, causing more attacks than other treatments. Perhaps the attractive cue of the ethanol-treated bolts is much stronger than the repellent cues produced by verbenone or verbenone and methyl salicylate combined. Frühbrodt et al. (2024) concluded that the ecology and function of verbenone are poorly understood and should be thoroughly studied before being used in pest management. Perhaps ambrosia beetle adults bore multiple times after landing on bolts with verbenone and methyl salicylate, considering bolts unsuitable for colonization. After adult *X. crassiusculus* attack, there was a mismatch between the number of entry holes and galleries formed inside the wood (Cambronero-Heinrichs et al. 2024). However, many studies showed that verbenone, combined with methyl salicylate, reduced the number of ambrosia beetles collected in traps but reported no difference in tree attacks (Ranger et al. 2021). Thus, more research is warranted to understand the driving factors associated with the interaction between adult *X. crassiusculus* and verbenone.

As shown in many studies (Frank and Sadof 2011, Reding et al. 2013, Ranger et al. 2016, Williamson et al. 2023), permethrin effectively reduced ambrosia beetle attacks when ethanol was added to cored bolts. As ethanol concentrations increased from 10% to 50%, ambrosia beetle attacks increased on bolts. In this study, permethrin effectively reduced ambrosia beetle attacks, regardless of the concentration of ethanol infused in bolts. Permethrin elicits contact or noncontact repellency in arthropods, but studies show mixed reports (Dobrin and Hammond 1985, Lowery and Boiteau 1988, Lin et al. 1993, Eisen et al. 2017, Joseph 2018). Ambrosia beetle adults approached permethrin-treated bolts but did not bore into those bolts (Williamson et al. 2023). This suggests that permethrin likely elicited contact repellency, or adults were moribund or died after encountering the treated bolts. For immediate use, data suggest that preventative trunk application of permethrin is still the best management tactic for ambrosia beetles in ornamental nurseries. More research is needed to understand if the maximum label rate of permethrin is indeed needed for ambrosia beetle management. Perhaps reduced label rates of permethrin could provide adequate ambrosia beetle control and help reduce the risk of exposure to non-targets, especially pollinators in ornamental nurseries.

In summary, results showed that verbenone alone or combined with methyl salicylate did not reduce ambrosia beetle attacks compared to permethrin. Permethrin effectively reduced ambrosia beetle attacks on young trees in ornamental nurseries. *X. crassiusculus* is the major species in the current study, although some adult *X. germanus* were also recovered from bolts. Because verbenone has shown promise against other adult ambrosia beetles, such as *X. glabratus* in other regions or systems (Hughes et al. 2017, Rivera et al. 2020, Agnello et al. 2021), the properties and ecology of verbenone should be carefully studied to refine repellent properties under varied environmental conditions and commodity systems. Research should also seek alternatives to permethrin trunk sprays to minimize negative impacts on non-targets, including pollinators and biological control agents, in ornamental nurseries.
